# Editorial

**Published:** 1876-03

**Authors:** 


### Editorial.

### A SEVERE CASE.

A gentleman who has been in practice for several years describes the following case and asks for advice.

He says: "A young lady called on me for advice about a tooth she had that had been treated—and I think shamefully too—by one claiming to be a dentist. The pulp had been exposed; he applied arsenious paste to devitalize it; this was done in such a careless manner as to destroy much of the soft tissue about the tooth. The gum had so sloughed off, before I saw it, as to expose the process between the tooth affected and the first bicuspid—and its neigbor, the second bicuspid.

The dentist had crowded a pledget of cotton saturated with arsenious paste, not into the cavity of decay, but down between the teeth; this had been there about two days; I removed it and did all I could to subdue the inflammation. The tooth was not as sore to the touch as one would be inclined to think.

Ater some time thc soreness abated, when I filled the root with Guilloi's cement and have been waiting for the tooth to become more firm in its place, as it is quite loose, and does not seem to come in contact with the process at all; this has been the condition of the tooth for about three months, and it seems as loose to-day as when first presented. Now what shall I do with the case?

What treatment is best in periodontitis?" M.

Now it is not an easy matter to decide what advice to give or what suggestions to make in regard to this case. But one thing we will suggest that Dr. M., should, as soon as possible, find the man who did this mischief and if he is a scoundrel, make an honest man of him, if possible, or if stupid ignorance is his disease, then enlighten him a little, enough at least that he will not commit such a blunder again.

But now what shall or can be done for this case; it is impossible to decide without a personal inspection. It may be that the loss of the tooth is unavoidable, this will depend very much upon the constitution and susceptibility of the patient, and upon the extent of the poisoning. If the patient is feeble, the blood poor in quality and the recuperative power deficient, the case is a discouraging one, so far as the pseservation of the tooth is concerned at least. But if the opposite conditions exist there is ground for hope; and it may be that much might be gained by systemic treatment, such as freeing it from any poisonous or deleterious agent or influence that may be present, and then toning up, invigorating and nourishing treatment will add vastly to the probabilities of success.

The local condition should receive attention. There is probably more or less necrosis of the alveolus; and so far as this exists it should at once be wholly removed, for it will be impossible to bring about any reparation until this is done. The periosteum can not assume a healthy condition while there is dead bone in contact with it.

The proper removal of necrosed alveolus is not always easily accomplished; exfoliation does not always take place in necrosis of the alveolus and the removal of the dead part and that only, is sometimes attended with difficulty. But in connection with excision some solvent—elixer vitriol, for instance, may be used with great benefit; thus dissolving the dead and stimulating the living. The persistent loose condition of the tooth and the chronic thickened state of the periosteum is indicative of dead process. After the removal of the latter the former will rapidly return to comparative health, and a reparation of the whole surrounding structure, be effected in a short time. But if there is a disposition to sloughing by the soft parts after this treatment, then systemic building up is surely indicated and, with this local treatment may be employed, embracing the following principles, viz: The removal and exclusion of irritants, by disinfectants and antiseptics, stimulants; if increased action is necessary, counter-irritants; if there is an excess of blood in the parts, escharotics, if need be, to retard and arrest discharge; and with all maintain good nutrition.

The treatment involved in these suggestions will, if faithfully and intelligently carried out, secure good results in almost any case.

---

### ABRASION OF THE TEETH, WITH SENSITIVENESS.

Dr. S., in writing says, "I have a case to be treated which puzzles me, and about which I take the liberty of consulting you.

The patient is a married lady about twenty-eight years old, quite healthy apparently. The upper central incisors have furrows on the labial surfaces near the festoons of the gums. The groove on the left central is much more plainly defined than the one on the right. In neither, however, do they penetrate entirely through the enamel.

These grooves have not been formed apparently by the coalescing or running together of pits in the enamel, indeed they are so smooth that they appear like simple depressions in the enamel—natural congenital deformities.

She complains of great sensitiveness in the furrows, especially when she brushes her teeth. She has used soap for cleansing, also prepared chalk. Will soap have a tendency to aggravate this affection? How shall I treat it? Is it advisable to use nitrate of silver and remove the stain by grinding? I have tried iodine. Is it a case of electrolisis?"

Every dentist has, perhaps, found just such cases.

Well how shall they be treated? Quite a step will have been made when the cause of this affection shall have been definitely discovered.

In the first place, they do not occur from the use of the brush, nor from the use of soap, nor from chalk, nor does galvanic action play any important part in its production.

There is usually found in connection with this affection, an abnormal condition of the mucous membrane covering the margin of the gums and consequently a vitiated secretion. This may be an irritant to the dentine, at the time of its exudation, or very soon become changed to that extent.

Attention in all such cases should be given to the gum, and when it is restored to a healthy condition and the oral secretion restored to a normal condition, there will be little trouble with sensitive dentine as described above.

We have in no instance found much sensitiveness before the exposure of the dentine. One is liable to be deceived as to whether the enamel is entirely penetrated, from the fact that the grooves throughout are as smooth and polished, and apparently as dense as the enamel upon the surface.

Local treatment directed to the sensitive dentine only, will avail little or nothing, except the tissue is devitalized and this most assuredly should not be done. Nitrate of silver, chloride of zinc or any kindred escharotic may be applied, but they will give no permanent relief, except so far as they may devitalize. Accomplishing this superficially, will generally give relief from the inconvenience, for a time at least, or so long as protection is afforded by the devitalized strata; beneath this protection restoration may take place. In some cases the affection is confined to a mere stratum, then a superficial devitalization, would give relief and if the cause of the affection was removed the relief would be permanent.

The use of nitrate of silver as Dr. S., proposes, would be productive of benefit only in very exceptional cases.

These grooves when they have at all penetrated the dentine should be properly formed and filled; if they are very sensitive after excavation they should be filled with a non-conductor—Hill's stopping perhaps—till recuperation takes place, then fill with gold.

### MICHIGAN UNIVERSITY DENTAL DEPARTMENT.

The Dental College, which is in its incipiency, is meeting with very marked success. Far more interest is being manifested in it in all directions than was anticipated, or perhaps than those immediately connected with it had a right to expect, but encouragement comes from every quarter. Those in authority in the University have manifested the warmest interest, and have given every facility in their power for carrying forward the work. The dental profession of Michigan has also shown great interest in the success and prosperity of the new enterprise, evidences of which were shown by the fostering care of the State Dental Society, manifested by liberal contributions, the appointment of a visiting and consulting committee, etc. About thirty volumes were recently contributed by Dr. Spellman, of Detroit. Drs. Thomas, of Detroit, and Finch, of Adrian, have been present and rendered valuable aid. Within a few days Dr. Robinson, of Jackson, spent a day or two in the college, and manifested his interest by taking part in the clinics. He also gave a lecture before the class, which received close attention. It was full of excellent suggestions and advice, and will certainly not soon be forgotten by those who heard it. In every respect the Dental College of Michigan University is proving a great success far beyond the expectation of its originators.

### IOWA STATE DENTAL SOCIETY.

The annual meeting of the Iowa State Dental Society will be held on Tuesday the 16th of May, 1876, at Burlington.

It is very desirable that the profession of the state be present so far as possible, and indeed all are most cordially invited to be present and participate in the proceedings.

J. P. Wilson, Sec.

The Iowa Society is an active and efficient one. It has been in operation for many years and has been the means of great good to the profession in that state.

The profession throughout the state should make it a point to be present and each one be ready to add his contribution to make the proceedings interesting and effective.

We hope that this year all the state societies will be more fully attended than ever before.

### MAD RIVER DENTAL SOCIETY.

The annual meeting of the Mad River Valley Dental Association will be held in the Beckel House Parlor, Dayton, Ohio, on Tuesday, April 4th, 1876, commencing at 10 o'clock

TOPICS FOR DISCUSSION.

1. Sensitive Dentine, how it may be best overcome?
2. Idiosyncrasies of the patient to be considered as to success or failure in filling teeth.
3. What success are we meeting with in preserving exposed nerves?
4. Our diet in its relation to the teeth.
5. Derangement of the organism consequent upon diseased teeth.
6. Relative merits of rubber and celluloid as bases for artificial teeth.

Essays are expected from Drs. Bradley, Sample, Whiteside, Sabine, Stipp and Geo. L. Paine.

S. B. Tizzard, E. F. Sample, J. H. Paine, Executive Committee; A. J. Grosvenor, Sec'y; Rob't. Corson, Pres.

